# Cystitis Cystica Et Glandularis Causing Lower Urinary Tract Symptoms in a 29-Year-Old Male

**DOI:** 10.7759/cureus.17144

**Published:** 2021-08-13

**Authors:** Young Son, Ian Madison, Julia Scali, Paul Chialastri, Gordon Brown

**Affiliations:** 1 Urology, Rowan University School of Osteopathic Medicine, Stratford, USA; 2 Urology, Philadelphia College of Osteopathic Medicine, Philadelphia, USA; 3 Urology, New Jersey Urology, Voorhees, USA

**Keywords:** cystitis cystica, cystitis cystica glandularis, urology, lower urinary tract symptoms, pathology

## Abstract

We present the case of a 29-year-old male who presented to the office with three years of persistent lower urinary tract symptoms and hematuria. On workup, the patient was determined to have an atypical condition contributing to his symptoms. There are numerous causes of lower urinary tract symptoms that can occur in young men. These symptoms, including frequency, urgency, dysuria, and hematuria, are typically caused by common benign conditions such as urethritis, urolithiasis, and urinary tract infections. Prostatic pathology does not typically manifest in this population. Likewise, a more serious condition such as a mass or carcinoma may contribute to persistent lower urinary tract symptoms and hematuria less often in young men. In our patient, a benign mass later identified as cystitis cystica et glandularis was discovered to be contributing to his reported discomfort.

## Introduction

Cystitis cystica et glandularis is most often encountered as an incidental finding and has a predilection for the trigone area of the bladder. Grossly, it may appear as a raised nodular lesion with an intact urothelial surface. Microscopically, the cysts are well-defined nests of urothelium resembling von Brunn’s nests, which are nests of benign urothelial cells in the lamina propria, but with central cystic dilation of the lumen lined by luminal cuboidal and columnar cells [1].

Von Brunn’s nests are one of the most common lesions in the lower urinary tract. Up to 60% of bladders analyzed postmortem showed evidence of cystitis cystica et glandularis, most commonly in the bladder neck and trigone [2]. For unknown reasons, the urothelium undergoes metaplasia into cuboidal or columnar cells, with or without goblet cells, which can irritate the bladder in some individuals. On rare occasions, a congregate of goblet cells can cause lesions as well as lower urinary tract symptoms.

Risk factors for developing the disorder include long-standing inflammation from chronic irritation such as infection, stones, or indwelling catheters [3]. There have been reported cases of cystitis cystica et glandularis in children and women; however, cases are rarer in young men.

## Case presentation

A 29-year-old male with no known past medical history was referred to the office for lower urinary tract symptoms and hematuria. He described a three-year history of symptoms including decreased force and caliber of his stream, difficulty urinating, and intermittent gross hematuria. He denied any smoking history, fever, dysuria, or flank pain. A urine sample was submitted for urinalysis, which was negative for signs of infection, and urine culture, which was also negative. He was treated with tamsulosin and was advised to return in two weeks for evaluation with in-office cystoscopy if the symptoms persisted.

The patient returned to the office two weeks later with persistent lower urinary tract symptoms. A repeat urine sample was submitted for analysis the prior day, which was again negative. He then underwent in-office cystoscopy which revealed an inflammatory-appearing mass at the bladder neck and trigone and base of the prostate gland. The urethra was normal without strictures. A computerized tomography (CT) urogram was ordered to further evaluate for any lesions due to hematuria. Transurethral resection of the bladder tumor was scheduled for the operating room.

**Figure 1:**
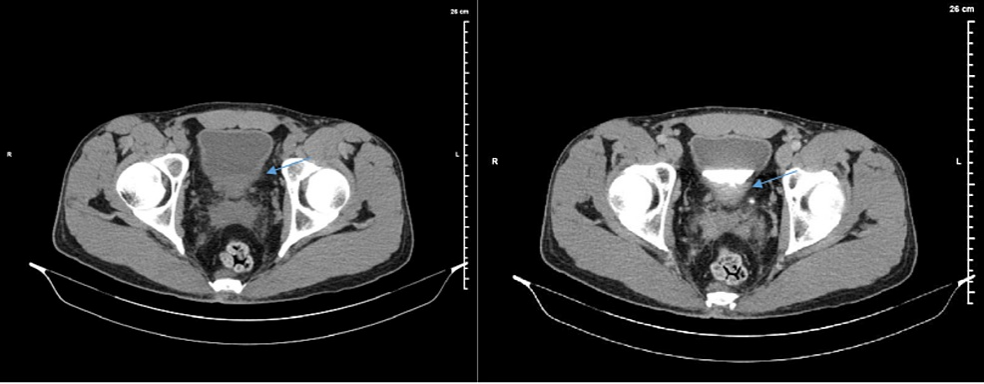
Left: Noncontrast CT transverse image of the bladder. Blue arrow is pointing at the thickened posterior bladder wall. Right: CT urogram, nine minutes delayed transverse image of the bladder. Blue arrow is showing posterior wall enhancement. CT: computerized tomography

**Figure 2:**
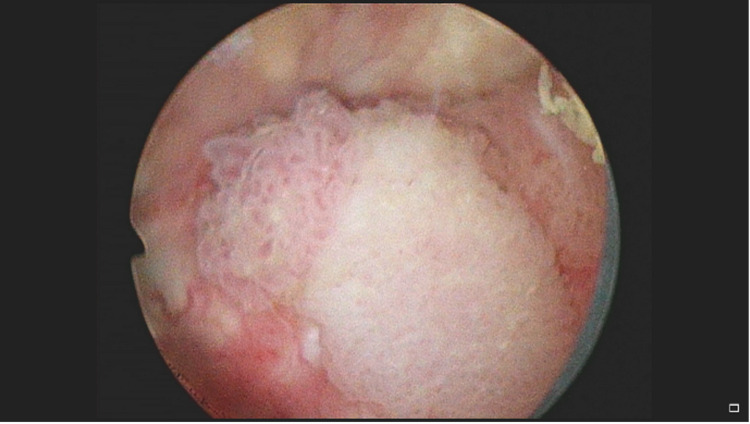
Intraoperative cystoscopy at the bladder neck showing a cystic mass-like structure obstructing the bladder outlet.

**Figure 3:**
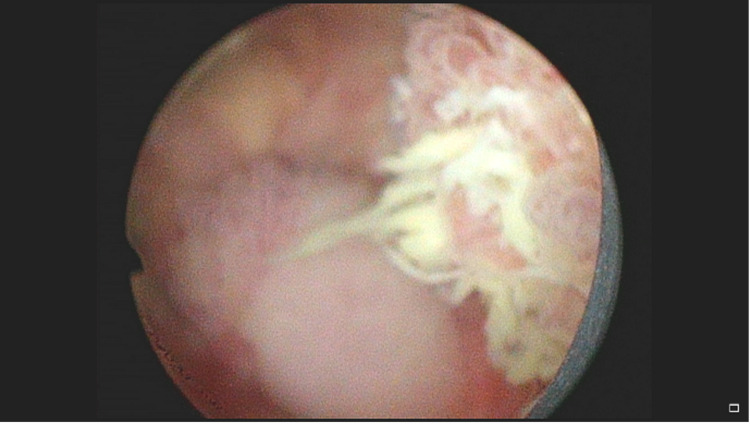
Intraoperative cystoscopy showing a mass with yellowish appearance and cystic structures.

**Figure 4:**
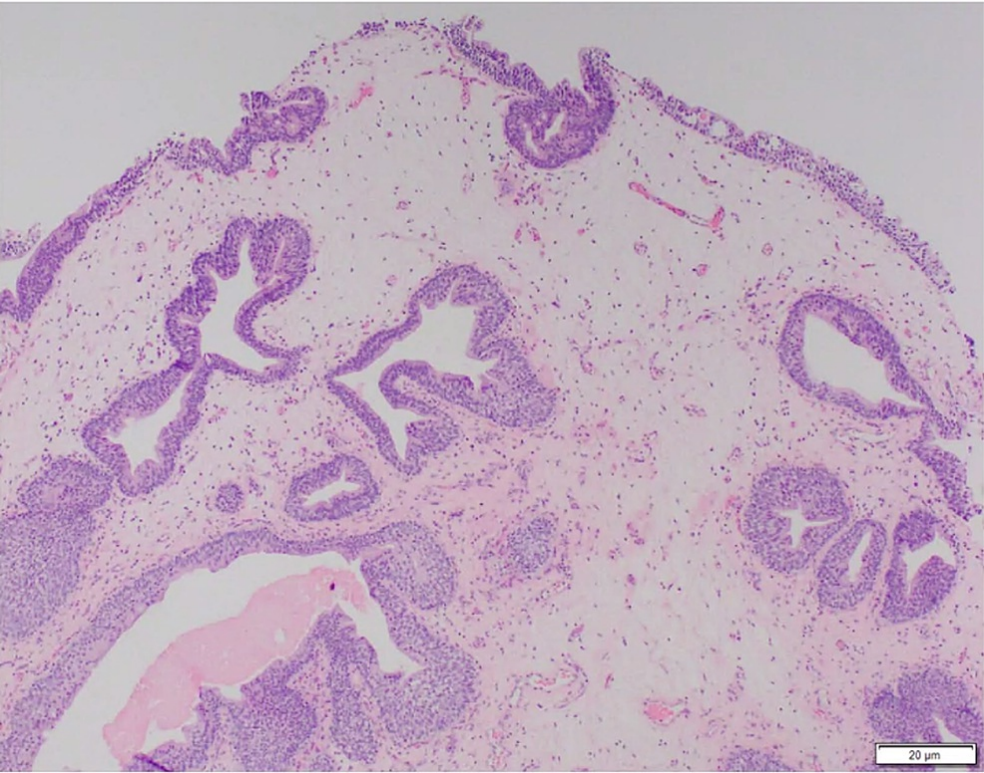
Pathological examination of the urothelium with florid cystitis cystica et glandularis. Focal muscularis present in the specimen, with no evidence of carcinoma.

Outpatient CT urogram was significant for posterior wall thickening with low levels of enhancement without any lesions (Figure 1). Two weeks after undergoing CT evaluation, the patient returned to the operating room for cystoscopy. Cystoscopy in the operating room showed cystic lesions at the level of the prostate extending to the trigone of the bladder (Figures 2, 3). The mass was partially obstructing the prostatic urethra. Transurethral resection of the tumor was performed, and samples were sent for pathology. Although pathology examination was negative for carcinoma, it demonstrated urothelium with florid cystitis cystica et glandularis with adequate tissue sampling proven by the presence of focal muscularis propria (Figure 4).

During his postoperative follow-up in the office, the patient reported resolution of his voiding symptoms. Urinalysis demonstrated microscopic hematuria, likely due to the recent procedure, and no evidence of infection. Due to the posterior wall enhancement seen on the CT urogram, a discussion was held regarding the potential need for re-resection and biopsy. Meanwhile, the patient reported significant alleviation of his voiding symptoms after elimination of the mass at the base of his prostate.

## Discussion

There are numerous causes of lower urinary tract symptoms that can occur in young men. Exceedingly common causes of lower urinary symptoms and hematuria in young men include benign prostatic hyperplasia, sexually transmitted diseases, and urinary tract infection. After routine differentials have been ruled out, rare conditions such as cystitis cystica et glandularis must be included. In our patient, a mass was discovered on in-office cystoscopy adjacent to the prostatic urethra. The mass could have been acting as a ball-valve, causing obstruction and decreased caliber of stream, hematuria, and incomplete emptying.

Cystitis cystica et glandularis is considered to be a hyperproliferative disorder; however, it has not yet been shown to be a precursor to malignant lesions. According to previously reported cases, it has been found to coexist with concurrent bladder carcinoma [4]. Several retrospective studies have demonstrated no link between the diagnosis of cystitis cystica et glandularis and the eventual development into adenocarcinoma of the bladder [5]. The patient was instructed to follow up one month after the postoperative appointment to track the alleviation of lower urinary tract symptoms and to repeat a urinalysis. No active surveillance was planned as cystitis cystica et glandularis was not deemed to be premalignant.

Microscopic pathologic evaluation is necessary to confirm the diagnosis of cystitis cystica et glandularis as gross pathology can resemble malignancy. There are two subtypes of cystitis cystica et glandularis. The typical type is a cystically dilated structure with luminal cuboidal or columnar cells surrounded by urothelial cells. The intestinal type is similar to the typical type, but with the presence of goblet cells that secrete mucous [6]. The differential for bladder wall thickening based on gross histology and CT imaging is broad and includes urothelial carcinoma, cystitis, cystitis cystica et glandularis, urothelial polyps, and other benign and malignant tumors [7].

Bladder wall thickening seen on CT urogram is consistent with cystitis cystica et glandularis compared to malignancy, especially without a significant smoking history. A trial of anticholinergic medication and treatment of the underlying cause of symptoms is the first-line treatment for cystitis cystica et glandularis. Although cystoscopy should not be the initial diagnostic modality, it should be considered if symptoms persist and other causes are ruled out. In patients with symptomatic cystitis cystica et glandularis, refractory to medication, resection of the lesion is the treatment of choice for alleviating symptoms. Other options for treatment include postoperative intravesical instillation of pirarubicin [8].

## Conclusions

We described a rare presentation of a young man with cystitis cystica et glandularis that caused lower urinary tract symptoms. Although a much less common reason for causing long-term lower urinary symptoms and hematuria, cystitis cystica et glandularis must be in the differential diagnosis, especially when workup for other causes are unrevealing.
